# Appropriate LDL-C-to-HDL-C Ratio Cutoffs for Categorization of Cardiovascular Disease Risk Factors among Uygur Adults in Xinjiang, China

**DOI:** 10.3390/ijerph13020235

**Published:** 2016-02-19

**Authors:** Qing-Jie Chen, Hong-Mei Lai, Bang-Dang Chen, Xiao-Mei Li, Hui Zhai, Chun-Hui He, Shuo Pan, Jun-Yi Luo, Jing Gao, Fen Liu, Yi-Tong Ma, Yi-Ning Yang

**Affiliations:** 1 Department of Cardiology, First Affiliated Hospital of Xinjiang Medical University, Urumqi 830054, China; chenqj0920@163.com (Q.-J.C.); lixm505@163.com (X.-M.L.); zhaihui1985@126.com (H.Z.); hechunhuiyisheng@163.com (C.-H.H.); parker1985@sina.com (S.P.); luojunyi3130@126.com (J.-Y.L.); gaojing1110@126.com (J.G.); 2 Xinjiang Key Laboratory of Cardiovascular Disease Research, Urumqi 830054, China; chenbangdang@126.com (B.-D.C.); fenliu82@163.com (F.L.); 3 Department of Cardiology, People’s Hospital of Xinjiang Uygur Autonomous Region, Urumqi 830001, China; 15999168372@139.com; 4 Clinical Research Institute of Xinjiang Medical University, Urumqi 830054, China; 5 Department of Cardiology, People’s Hospital of Shaanxi Province, Xian 710000, China

**Keywords:** cutoff, LDL-C-to-HDL-C ratio, cardiovascular disease risk factors, Uygur adults, Xinjiang

## Abstract

Elevated LDL-C/HDL-C ratio has been shown to be a marker of lipid metabolism as well as a good predictor of coronary artery disease (CAD). Thus, the aim of this study was to investigate whether the LDL-C/HDL-C ratio is useful for detecting cardiovascular disease (CVD) risk factors in general healthy Uygur adults in Xinjiang. A total of 4047 Uygur subjects aged ≥35 years were selected from the Cardiovascular Risk Survey (CRS) study which was carried out from October 2007 to March 2010. Anthropometric data, blood pressure, lipid profile and fasting glucose were measured in all participants. The prevalence, sensitivity, specificity and distance on the receiver operating characteristic (ROC) curve of each LDL-C/HDL-C ratio were calculated. The prevalence of high LDL-C and low HDL-C cholesterol was high and positively correlated with higher LDL-C/HDL-C ratio in the Uygur population. In both men and women, we detected a slight apparent trend of high prevalence of hypertension and hypercholesterolemia with higher LDL-C/HDL-C ratio. Our study also demonstrated that the discriminatory power of the LDL-C/HDL-C ratio for CVD risk factors was slightly stronger in men than in women. Analysis of the shortest distance in the ROC curves for hypertension, dyslipidemia, diabetes, or ≥two of these risk factors suggested a LDL-C/HDL-C ratio cutoff of 2.5 for both men and women. The results of this study showed that a LDL-C/HDL-C ratio cut-off of 2.5 might be used as the predictive marker to detect CVD risk factors among Uygur adults in Xinjiang.

## 1. Introduction

The increasing prevalence of coronary artery disease (CAD) is emerging as the major cause of mortality in China, with the total number of people dying of CAD reaching one million each year in China. In Xinjiang, the prevalence of CAD in the Uygur population was 24.2% in 2012, which was much higher than the average prevalence of CAD (7.2%) in China. Thus, early detection and effective treatment for preventing CAD progression in Uygur population is necessary.

Low-density lipoprotein cholesterol (LDL-C) is a well-established risk factor for CAD [1,2,3]. Lowering LDL-C has been a cornerstone in the treatment and prevention of atherosclerosis. Intensive statin therapy has been shown to significantly decrease the occurrence of cardiovascular disease (CVD) and mortality in patients with CAD [1]. However, residual cardiovascular risk still occurs in some patients despite achievement of LDL goals with statin therapy. The residual cardiovascular risk is complex, partly due to atherogenic dyslipidemia, which is characterized by lower levels of high-density lipoprotein and elevated plasma triglyceride concentrations [4]. Low high-density lipoprotein cholesterol (HDL-C) is an established coronary risk factor that is independent of LDL-C level, and is inversely associated with cardiovascular events.

Recently, the LDL-C to HDL-C (LDL-C/HDL-C) ratio has been demonstrated to be a surrogate marker of lipid-lowering therapies associated with coronary heart disease (CHD) [5]. Studies have shown that LDL-C/HDL-C ratio was a more precise predictor for cardiovascular events and their relative risk [6,7,8] and it was positively associated with percentage volume changes of coronary plaque burden, high percentage of lipid volume and a low percentage of fibrous volume in LMCA lesions [9,10,11,12,13,14]. Furthermore, it has been identified as a positive predictor for coronary lipid-rich plaque and plaque vulnerability in patients with chronic CAD [15]. The clinical use of LDL-C/HDL-C ratio and the cutoff points of LDL-C/HDL-C ratio are complicated by variations among ethnicities. It was important to apply the ethnically appropriate cutoff values of LDL-C/HDL-C ratio for assessing risk factors of CVD. In the present study, we investigated the associations between LDL-C/HDL-C ratio and risk factors for CVD in Uygur adults.

## 2. Methods

### 2.1. Study Design and Subject Recruitment

All participants were selected from the Cardiovascular Risk Survey (CRS) study, aged from 35 to 88 years and recruited between 2007 and 2010. Full details of the CRS study have been published elsewhere [16]. The study was approved by the Ethics Committee of the First Affiliated Hospital of Xinjiang Medical University and was conducted in accordance with the standards of the Declaration of Helsinki (Ethics Approval Number:20080724). All participants signed the informed consent form. In brief, it is a cross-sectional study of risk factors for CVD in the multiethnic population (mainly Han, Uygur and Hazakh population) in Xinjiang which was performed from June 2007 to March 2010, and it applied a 4-stage stratified sampling method to choose a representative sample from the general population in Xinjiang, which is located in the northwest of China. All participants completed the questionnaire and physical examination components of the baseline assessment. Individuals were excluded from this study if they have incomplete data, a history of CAD and cerebral vascular disease, heart failure, stroke, electrocardiographic signs of CAD, regional wall motion abnormalities, and relevant valvular abnormalities in echocardiograms and/or carotid atherogenesis [2]. A total of 4047 Uygur participants, including 1743 males and 2304 females, with complete data were enrolled in our study.

### 2.2. Baseline Examination and Laboratory Tests

During the interview, all subjects finished a standard questionnaire regarding individuals’ information, including lifestyle (including smoking and drinking), medical history and physiological conditions. Blood pressure, height and weight were measured, and fasting blood samples were taken from all participants. All information was collected by trained research staff and entered into a centralized electronic database. Body weight was measured while the subjects were without clothes or shoes with a double balance placed on a firm surface. Height was measured using calibrated height meters while subjects stood erect and barefooted, with feet placed together and looking forward. BMI was calculated by dividing the weight in kilograms by the height in meters squared.

Blood pressure was measured three times using a mercury sphygmomanometer in the sitting position after a 10 min rest, and all subjects were required to refrain from smoking or consuming caffeine 30 min prior to measurement. Fasting peripheral blood samples were obtained from all participants for the assessment of routine biochemical variables. All collected samples were immediately placed and stored on dry ice, transported to Xinjiang Coronary Artery Disease VIP Laboratory. The total cholesterol, triglyceride, low density lipoprotein (LDL-C), high density lipoprotein (HDL-C) and fasting blood glucose were measured in the Clinical Laboratory Department of the First Affiliated Hospital of Xinjiang Medical University by the Biochemical Analyzer (Dimension AR/AVL Clinical Chemistry System, Newark, NJ, USA) [3,17].

### 2.3. Definition of Risk Factors

Risk factors were estimated on the basis of a questionnaire administered at the baseline visit, physical examination, and laboratory tests. Self-reported regular tobacco use in the previous 6 months was considered as current smokers. Hypertension was defined as history of hypertension and/or an average systolic blood pressure ≥140 mmHg and/or an average diastolic blood pressure ≥90 mmHg. Participants were considered diabetic if they reported a physician diagnosis of diabetes or were taking anti-diabetic medication or had fasting/non-fasting glucose ≥126 mg/dL/≥200 mg/dL.

Hypercholesterolemia was defined by the concentration of total cholesterol concentration >6.22 mmol/L (240 mg/dL), and hypertriglyceridemia was defined by the concentration of triglyceride >2.26 mmol/L (200 mg/dL). A LDL-C concentration of >4.14 mmol/L (160 mg/dL) was defined as high LDL-C, and a HDL-C concentration of <1.04 mmol/L (40 mg/dL) was defined as low HDL-C [4]. Dyslipidemia was defined as the existence of at least one of the four abnormal lipid concentrations mentioned above, or self-reported use of antihyperlipidemic medication.

### 2.4. Statistical Analysis

All statistical analyses were performed with SPSS version 22.0 for Windows (SPSS Inc., Chicago, IL, USA). All statistical assessments were two-sided, and a *p* value <0.05 was considered statistically significant. Distributions of risk factors measured in the study participants were expressed using gender-specific mean values ± standard deviation for continuous variables and gender-specific proportions for discrete data, respectively. One-way ANOVA was applied for comparing differences among different LDL-C/HDL-C groups. Age-standardization was performed via the direct method according to the 2000 Xinjiang Uygur population as the standard population. Dichotomous variables were generated to determine the sensitivity and specificity of each LDL-C/HDL-C ratio for the detection of hypertension, dyslipidemia, diabetes, and two or more of these risk factors. Additionally, a receiver operating characteristic (ROC) curve was established to determine the optimal cutoff value for predicting the risk factors. The distance on the ROC curve of each LDL-C/HDL-C ratio value was calculated as the square root of [(1 − sensitivity)^2^ + (1 − specificity)^2^]. The LDL-C/HDL-C ratio value with the shortest distance on the ROC curve was considered as the appropriate cutoff point.

## 3. Results

### 3.1. Age-Standardized CVD Risk Factors in Uygur by LDL-C/HDL-C Ratio Category

The distribution of sample size associated with age and gender is shown in Table 1. Basic lipid profiles, blood pressures and their age-related distributions are shown in Table 2. In both men and women, LDL-C/HDL-C ratio increased with the increase of LDL-C concentration and the decrease of HDL-C concentration. Such correlation was not observed when LDL-C/HDL-C ratio exceeds 5.0. In addition, we failed to observe a significant correlation between LDL-C/HDL-C ratio and any of the following factors: systolic or diastolic blood pressure, and the concentration of total cholesterol, triglyceride, or fasting glucose (Table 2).

### 3.2. Prevalence of Risk Factors in Uygur by LDL-C/HDL-C Ratio Category

We demonstrated a positive correlation between low HDL-C values and high LDL-C/HDL-C ratio in both men and women (Table 2). In addition, we also observed a positive correlation between high LDL-C values and high LDL-C/HDL-C ratio in both men and women. The prevalence of hypertension and hypercholesterolemia has a slightly increasing trend with higher LDL-C/HDL-C ratio in both men and women. (Table 3). Furthermore, we investigated the distribution of LDL-C/HDL-C ratio in healthy subjects and those showing CVD risk factors, which is shown in Table 4. The percentage of subjects with CVD risk factors increases with the increase of LDL-C/HDL-C ratio. When LDL-C/HDL-C ratio reached 2.5, almost all subjects had at least one CVD risk factor, such as hypertension, diabetes, or dyslipidemia.

### 3.3. Sensitivity, Specificity, and Distance in the Receiver Operating Characteristic (ROC) Curve for LDL-C/HDL-C Ratio Cutoff Points in Uygur

**Figure 1:**
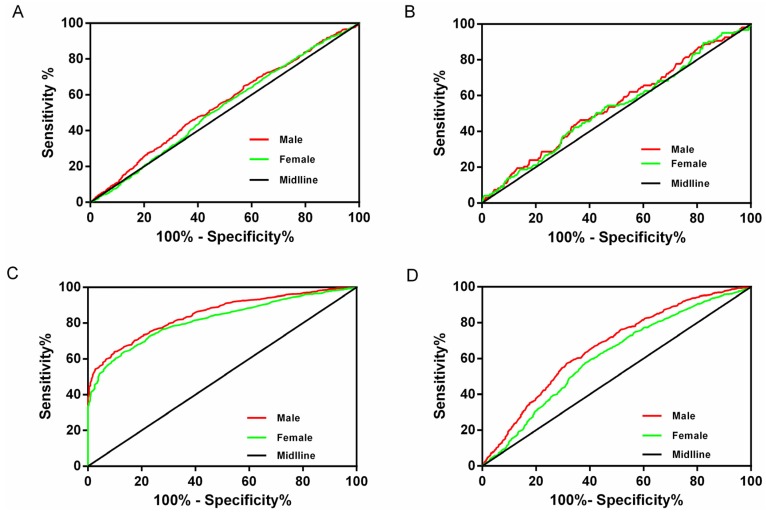
ROC curves to detect CVD risk factors in males and females.(**A**) ROC curves to detect hypertension in males and females; (**B**) ROC curves to detect diabetes in males and females; (**C**) ROC curves to detect dyslipidemia in males and females; (**D**) ROC curves to detect ≥2 of these risk factors in males and females.

To identify an appropriate LDH-C/HDL-C cutoff point, we constructed the ROC curves for men and women (Figure 1). The population percentile of each LDL-C/HDL-C ratio level and the sensitivity, specificity, and distance on the ROC curve for the detection of hypertension, dyslipidemia, diabetes, and ≥two of these risk factors are presented in Table 5 for men and women, respectively. In men, the cutoff point was 2.0 for dyslipidemia and 3.0 for diabetes. The shortest distances on the ROC curve for hypertension and ≥two of these risk factors was 2.5 for both men and women. In women, the shortest distances on the ROC curves were similar for hypertension, diabetes and ≥two of these risk factors and the cutoff point for these three risk factors was 2.5, while the cutoff for dyslipidemia was 2.0. The LDL-C/HDL-C ratio of 2.5 appeared to be the optimal cutoff value in women. The AUCs of each cardiovascular risk factor in men and women are summarized in Table 6. Our data suggested that the discriminatory value of LDL-C/HDL-C ratio for CVD risk factors was slightly stronger in men than in women.

## 4. Discussion

In the present study, we determined the optimal cutoff value of the LDL-C/HDL-C ratio to evaluate CVD risk factors such as hypertension, diabetes, dyslipidemia, and ≥two risk factors in both men and women in the Uygur population in Xinjiang. This study provided the evidence that a higher LDL-C/HDL-C ratio was associated with an unfavorable cardiometabolic risk profile. The major findings of this study indicated that subjects with a LDL-C/HDL-C ratio greater than 2.5 had higher prevalence of CVD risk factors. The discriminatory power of the LDL-C/HDL-C ratio for CVD risk factors was slightly stronger in men than in women. To the best of our knowledge, this study was the first to analyze the accuracy of the LDL-C/HDL-C ratio in predicting CVD risk factors in the Uygur population in China. Previous studies reported that lipoprotein index can be a good predictor for CVD or coronary heart disease (CHD). Johnston *et al.* [5] showed that lipoprotein ratio was better than lipid protein to identify CAD patients, and the total cholesterol/HDL-C ratio had higher sensitivity and specificity for detecting CAD risk factors than total cholesterol or HDL alone. In another study, Lippi *et al.* [6] identified that LDL-C/HDL-C ratio was the most sensitive index among the lipid protein indices to detect mild severity CAD. However, there were few studies investigating the usefulness of the lipoprotein index, such as LDL-C/HDL-C ratio, to identify healthy subjects and subjects with CVD risk factors in a large population. One prior study showed that the LDL-C/HDL-C ratio was useful in assessing the risk of early stage atherosclerosis in Japanese type 2 diabetic patients [7]. Our study showed that the higher LDL-C/HDL-C ratio was significantly associated with higher prevalence of CVD risk factors in the Uygur population.

Dyslipidemia and insulin resistance played an important role in the pathogenesis of CVD risk factors, such as hypertension, diabetes and metabolic syndrome [18]. In our study, we analyzed the prevalence of cardiovascular risk factors in the Uygur population stratified by LDL-C/HDL-C ratio. We found that the prevalence of hypertension and dyslipidemia increased with the increase of LDL-C/HDL-C ratio, and the percentage of healthy subjects dropped sharply with the increase of LDL-C/HDL-C ratio. When LDL-C/HDL-C ratio reached 2.5, almost all subjects had at least one CVD risk factor, such as hypertension, diabetes, or dyslipidemia. Our results indicated that the LDL-C/HDL-C ratio might be a good index to distinguish people with or without CVD risk factors, which is in line with many other studies [6,7,8].

We identified that LDL-C/HDL-C ratio cutoffs for predicting CVD risk factors were the same in men and women. Our cut-off ratio was somewhat higher than that of Nicholls’s study [8], which showed that a LDL-C/HDL-C ratio of >2.0 was a risk factor for coronary artery plaque progression, and a LDL-C/HDL-C ratio of <1.5 was significantly related to plaque regression. However, the optimal cut-off ratio obtained from our study was lower than that obtained by Millan *et al.* [9]. They found that the cut-off values of LDL-C/HDL-C ratios for initiating primary prevention of CAD were >3.5 and 3.0 for men and women, respectively, and the cut-off ratios for initiating secondary prevention of CAD were 3.0 and 2.5 for men and women, respectively. Our cut-off ratio is inconsistent with those previous studies. Several factors may help to explain the discrepancies between studies. First, the participants included in Nicholls’s study were CHD patients, who were different from the participants of our study. It may help to explain why the cutoff of LDL-C/HDL-C ratio for predicting the progression of CVD was lower in Nicholls’s study. Second, race, life styles, living habits and living environments may contribute to these discrepancies. Third, genetic factors, especially the genes involved in obesity and lipid metabolism, such as PPAR and APOA5 genetic polymorphism [14,15,19], should be taken into consideration, because prior studies demonstrated that Europeans had higher incidence of metabolic syndrome associated with lipid metabolism [10,11,12,13,14,15,19]. Therefore, the optimal cutoff point of the LDL-C/HDL-C ratio may vary depending on a number of factors as mentioned above.

In the present study, our results showed that AUC of each cardiovascular risk factor associated with LDL-C/HLD-C ratio was significantly larger in men than in women, indicating that the LDL-C/HDL-C ratio may be a better predictor of CVD risk factors for Uygur men. The exact mechanisms underlying this phenomenon were unclear. Some factors should be taken into account. First, compared with Uygur women, Uygur men consumed more pasta, meat and milk products, which contain richer saturated fatty acids and trans-fatty acids, all of which leads to increased LDL concentration [20]. Second, Uygur men are usually heavy smokers and excessive drinkers, which may contribute to the dyslipidemia. Third, the differences in genetic factors involved in mediating the levels of lipid proteins between genders may play a certain role in the synthesis of LDL-C and HDL-C [18,21].

The current study has a number of strengths: (1) this was the first representative data collected from Uygur adults in Xinjiang. Therefore, these results may be extrapolated to adults of the Uygur population aged over 35 years; (2) we provided a wide range of LDL-C/HDL-C ratio values, which were stratified by sex, and also provide an optimal cutoff to distinguish healthy people and people displaying CVD risk factors. Thus, future studies may use the LDL-C/HDL-C ratios and cutoff values from our study to further explore the associated risk factors and intervention of dyslipidemia and metabolic syndrome in the Uygur adult population in Xinjiang. A number of limitations warrant mentioning. First, our study was a cross-sectional study which precludes the interpretability of cause-effect. Second, we were unable to exclude potential confounding effects of medication and the presence of other diseases on the concentration of lipid proteins due to inadequate information. Third, outliers and missing values of blood specimens were excluded from analysis, which might impact the outcomes of this study.

## 5. Conclusions

In conclusion, our study demonstrated that a LDL-C/HDL-C ratio of 2.5 is an optimal cutoff point to predict CVD risk factors in Uygur adults over 35 years old. Further studies are needed to investigate the predictive value of LDL-C/HDL-C ratio in the onset and progression of CHD over time.

## Figures and Tables

**Table 1:** The distribution of age and gender for all subjects in the study.

Sex/Age	35–39	40–44	45–49	50–54	55–59	60–64	65–69	70–74	75–79	80–84	85–89	Total
	Years	Years	Years	Years	Years	Years	Years	Years	Years	Years	Years	
Men	187	257	229	236	226	236	168	115	51	19	19	1743
Women	368	399	369	333	284	251	180	69	27	18	6	2304

**Table 2:** Age-standardized CVD risk factors in the Uygur population by LDL-C/HDL-C ratio category.

	LHR < 1.5	1.5 ≤ LHR < 2	2 ≤ LHR < 2.5	2.5 ≤ LHR < 3	3 ≤ LHR < 3.5	3.5 ≤ LHR < 4	4 ≤ LHR < 4.5	4.5 ≤ LHR < 5	LHR ≥ 5	*p* Value
**Men**										
Population distribution (%)	214 (11.4%)	378 (20.2%)	441 (23.5%)	361 (19.3%)	233 (12.4%)	139 (7.4%)	61 (3.3%)	36 (1.9%)	12 (0.6%)	
Systolic blood pressure (mmHg)	132.29 ± 20.04	131.18 ± 19.04	131.47 ± 19.34	132.09 ± 21.13	129.70 ± 17.68	132.57 ± 18.78	126.07 ± 16.27	133.37 ± 24.53	139.33 ± 29.58	0.285
Diastolic blood pressure (mmHg)	81.57 ± 14.36	80.54 ± 14.35	79.84 ± 13.84	81.27 ± 15.07	79.48 ± 14.19	81.89 ± 14.63	76.82 ± 13.10	78.91 ± 17.47	81.75 ± 23.40	0.29
Total cholesterol (mmol/L)	4.21 ± 1.03	4.34 ± 1.03	4.27 ± 1.10	4.25 ± 1.06	4.38 ± 1.11	4.42 ± 1.03	4.20 ± 0.97	4.25 ± 1.25	3.99 ± 0.96	0.44
HDL-C cholesterol (mmol/L)	1.63 ± 0.45	1.43 ± 0.38	1.26 ± 0.32	1.20 ± 0.30	1.06 ± 0.27	0.97 ± 0.26	0.87 ± 0.25	0.81 ± 0.27	0.62 ± 0.21	**<0.001**
LDL-C cholesterol (mmol/L)	2.05 ± 0.57	2.47 ± 0.64	2.78 ± 0.72	3.18 ± 0.78	3.35 ± 0.84	3.57 ± 0.97	3.58 ± 0.98	3.70 ± 1.26	3.16 ± 1.03	**<0.001**
Triglycerides (mmol/L)	1.60 ± 1.26	1.66 ± 1.25	1.58 ± 1.08	1.62 ± 1.23	1.54 ± 0.92	1.83 ± 1.59	1.69 ± 1.09	2.09 ± 3.14	1.89 ± 0.79	0.22
Fasting glucose (mmol/L)	4.91 ± 1.80	4.92 ± 1.71	4.81 ± 1.52	4.93 ± 1.98	4.79 ± 1.18	5.02 ± 1.94	5.80 ± 3.86	4.92 ± 1.08	4.92 ± 1.31	**0.027**
**Women**										
Population distribution (%)	273 (10.8%)	555 (21.9%)	603 (23.8%)	480 (19%)	279 (11%)	182 (7.2%)	84 (3.3%)	40 (1.6%)	35 (1.4%)	
Systolic blood pressure (mmHg)	131.78 ± 24	131.86 ± 21.21	132.10 ± 21.88	129.56 ± 21.81	128.45 ± 21.37	132.54 ± 24.96	132.67 ± 24.02	129.28 ± 24.22	129.4 ± 17.91	0.272
Diastolic blood pressure (mmHg)	80.98 ± 16.25	80.36 ± 14.98	80.05 ± 15.09	78.54 ± 15.09	77.91 ± 14.60	80.79 ± 16.85	79.49 ± 14.47	78.26 ± 15.24	77.8 ± 13.70	0.174
Total cholesterol (mmol/L)	4.34 ± 1.19	4.46 ± 1.10	4.34 ± 1.11	4.36 ± 1.11	4.31 ± 1.23	4.45 ± 1.05	4.53 ± 1.21	4.33 ± 1.03	4.64 ± 1.08	0.352
HDL-C cholesterol (mmol/L)	1.68 ± 0.45	1.43 ± 0.38	1.27 ± 0.32	1.14 ± 0.30	1.08 ± 0.30	0.99 ± 0.26	0.87 ± 0.25	0.78 ± 0.20	0.64 ± 0.22	**<0.001**
LDL-C cholesterol (mmol/L)	2.09 ± 0.58	2.46 ± 0.66	2.80 ± 0.70	3.04 ± 0.80	3.36 ± 0.94	3.60 ± 0.95	3.61 ± 1.03	3.70 ± 0.91	3.31 ± 1.13	**<0.001**
Triglycerides (mmol/L)	1.50 ± 1.01	1.61 ± 1.22	1.61 ± 1.16	1.60 ± 0.99	1.52 ± 0.94	1.62 ± 0.98	1.65 ± 1.07	1.62 ± 0.67	2.20 ± 1.44	0.075
Fasting glucose (mmol/L)	5.02 ± 1.65	4.92 ± 1.49	4.83 ± 1.21	4.92 ± 1.62	4.94 ± 1.52	4.74 ± 1.44	4.76 ± 0.93	4.64 ± 0.75	4.95 ± 1.23	0.45

Note: CVD: cardiovascular disease; LHR: LDL-C/HDL-C ratio.

**Table 3:** Age-standardized prevalence of risk factors in the Uygur population by LDL-C/HDL-C ratio category.

	LHR < 1.5	1.5 ≤ LHR < 2	2 ≤ LHR < 2.5	2.5 ≤ LHR < 3	3 ≤ LHR < 3.5	3.5 ≤ LHR < 4	4 ≤ LHR < 4.5	4.5 ≤ LHR < 5	LHR ≥ 5	*p* Value
**Men**										
Hypertension	36.4%	39.7%	43.1%	46.3%	50.6%	48.2%	44.3%	61.1%	33.3%	0.016
Diabetes	5.0%	6.1%	5.4%	6.8%	4.8%	8.8%	8.3%	5.9%	8.3%	0.85
Hypercholesterolemia	12.4%	14.5%	16.3%	16.1%	20.3%	19.1%	15.0%	20.6%	16.7%	0.53
High LDL cholesterol	2.8%	14.8%	33.1%	53.5%	63.1%	64.0%	67.2%	75.0%	50.0%	<0.001
Low HDL cholesterol	8.4%	13.8%	24.7%	32.4%	49.4%	64.0%	75.4%	91.7%	100.0%	<0.001
Hypertriglyceridemia	26.9%	34.2%	31.9%	31.7%	30.4%	36.0%	35.0%	35.3%	50.0%	0.57
**Women**										
Hypertension	35.9%	40.7%	43.3%	45.4%	45.2%	48.9%	40.5%	35.0%	37.1%	0.122
Diabetes	6.8%	6.1%	4.4%	4.5%	5.8%	2.8%	2.4%	0.0%	14.7%	0.049
Hypercholesterolemia	19.5%	19.6%	17.4%	17.8%	18.2%	19.3%	22.9%	23.7%	23.5%	0.904
High LDL cholesterol	5.1%	15.0%	32.8%	45.6%	59.9%	72.5%	67.9%	70.0%	57.1%	<0.001
Low HDL cholesterol	9.2%	14.1%	24.7%	38.3%	47.3%	60.4%	73.8%	92.5%	94.3%	<0.001
Hypertriglyceridemia	29.3%	30.3%	34.2%	35.4%	30.8%	33.1%	39.8%	44.7%	44.1%	0.168

Notes: LHR: LDL-C/HDL-C ratio.

**Table 4:** The distribution of risk factors stratified by LDL-C/HDL-C ratio category in healthy subjects and subjects with CVD risk factors.

LDL-C/HDL-C Ratio	LHR < 1.5	1.5 ≤ LHR < 2	2 ≤ LHR < 2.5	2.5 ≤ LHR < 3	3 ≤ LHR < 3.5	3.5 ≤ LHR < 4	4 ≤ LHR < 4.5	4.5 ≤ LHR < 5	LHR ≥ 5
Healthy subjects	37.7%	26.0%	13.6%	3.4%	0.0%	0.0%	0.0%	0.0%	0.0%
Subjects with CVD risk factors	62.3%	74.0%	86.4%	96.6%	100.0%	100.0%	100.0%	100.0%	100.0%

Notes: LHR: LDL-C/HDL-C ratio; CVD: Cardiovascular disease.

**Table 5:** Sensitivity (Sens), specificity (Spec), and distance in the receiver operating characteristic (ROC) curve for LDL-C/HDL-C ratio cutoff points in the Uygur population.

Odds Ratio	Proportion	Hypertension			Dyslipidemia			Diabetes			≥2 Risk Factors		
LDL-C/HDL-C Cutoffs	Percentile	Sens %	Spec %	Distance in ROC Curve	Sens %	Spec %	Distance in ROC Curve	Sens %	Spec %	Distance in ROC Curve	Sens %	Spec %	Distance in ROC Curve
**Men**													
1.5	12.3	89.9	14.2	0.87	94.6	31.7	0.69	90.9	12.3	0.88	95.4	16.8	0.83
2	33	70.2	36.3	0.70	79.6	69.8	0.36	70	33.2	0.73	80.6	41.4	0.62
2.5	57.1	46.9	60.5	0.66	56.6	95.6	0.44	49.1	57.1	0.67	59.2	66.5	0.53
3	75.7	27.5	78.4	0.76	32.8	100	0.67	28.2	75.8	0.30	35.4	82.2	0.67
3.5	87.2	14.1	88.3	0.87	17.3	100	0.83	18.2	87.4	0.83	18.6	90.6	0.82
4	94.6	5.8	95.2	0.94	7.2	100	0.93	7.3	94.8	0.93	7.8	96.2	0.92
4.5	97.9	2.2	98.1	0.98	2.8	100	0.97	2.7	98	0.97	3	98.5	1.00
5	99.5	0.4	99.4	1.00	0.7	100	0.99	0.9	99.5	0.99	0.6	99.6	0.99
**Women**													
1.5	10.79	90.10	13.60	0.87	93.90	28.20	0.72	85.70	11.80	0.89	94.00	15.30	0.85
2	32.71	68.20	37.10	0.71	77.90	69.50	0.38	57.10	34.30	0.79	76.70	41.10	0.63
2.5	56.54	44.00	60.70	0.68	54.40	94.10	0.46	38.90	58.40	0.74	53.70	65.40	0.58
3	75.50	23.20	78.50	0.80	30.40	100.00	0.70	21.40	77.50	0.82	29.40	81.70	0.73
3.5	86.53	12.40	87.30	0.89	17.20	100.00	0.83	9.50	87.20	0.91	15.60	89.10	0.85
4	93.72	5.00	93.80	0.95	7.80	100.00	0.92	5.60	94.30	0.95	6.40	94.70	0.94
4.5	97.04	2.20	97.00	0.98	3.70	100.00	0.96	4.00	97.40	0.96	3.00	97.50	0.97
5	98.62	1.00	98.70	0.98	1.60	100.00	0.98	3.20	98.90	0.97	1.50	99.00	0.99

Note: LHR: LDL-C/HDL-C ratio.

**Table 6:** AUC of each cardiovascular risk factor in men and women in the Uygur population.

AUC	Men (95% CI)	Women (95% CI)
Hypertension	0.544 (0.517, 0.572)	0.523 (0.499, 0.546)
Diabetes	0.543 (0.486, 0.599)	0.513 (0.478, 0.584)
Dyslipidemia	0.849 (0.831, 0.867)	0.818 (0.801, 0.835)
≥2 risk factors	0.663 (0.637, 0.689)	0.609 (0.586, 0.633)
